# P058. Refractory chronic migraine, fatigue and OnabotulinumtoxinA: a clinic setting experience

**DOI:** 10.1186/1129-2377-16-S1-A113

**Published:** 2015-09-28

**Authors:** Filippo Baldacci, Martina Cafalli, Cinzia Lucchesi, Sonia Mazzucchi, Elisa Dini, Ubaldo Bonuccelli, Sara Gori

**Affiliations:** Neurology Unit, Department of Clinical and Experimental Medicine, University of Pisa, Pisa, Italy

## Objective

To assess OnabotulinumtoxinA safety and efficacy in prophylactic treatment for chronic refractory migraine (headache occurring at least 15 days per month with lack of responsiveness to at least two preventive medications with established efficacy)[1] with associated fatigue symptom.

## Methods

From March 2014 to May 2015 patients meeting the clinical diagnostic criteria for chronic refractory migraine were enrolled. Patients were treated with OnabotulinumtoxinA every three months according to the standard procedure (155-195 units)[2]. At baseline (T0) and after 6 months, at the third treatment (T1), a structured questionnaire was administered, including: a) migraine features [frequency (headache days/month), pain severity (Verbal Numeric Scale, VNS), acute medicines consumption/month, disability (Headache Impact Test, HIT-6), ictal cutaneous allodynia (Allodynia Symptoms Check-list 12, ASC-12)]; b) associated symptoms [fatigue (Fatigue Severity Scale, FSS), anxiety symptoms (Generalized Anxiety Disorder, GAD-7), depressive symptoms (Patient Health Questionnaire, PHQ-9)]. Wilcoxon test was performed for the T0-T1 comparisons.

## Results

Twenty-one patients were enrolled (M/F=3/18; mean age: 52.6±9.71). A patient discontinued the study after the first treatment due to an adverse event (eyelid ptosis). Twenty patients were evaluated at T1, with migraine features changing as follow: T0 frequency Me=30 IQR=10, T1 frequency Me=13 IQR=13, T0 VNS Me=8 IQR=3, T1 VNS Me=8 IQR=4, T0 acute medicines consumption/month Me=20 IQR=15, T1 acute medicines consumption/month Me=7 IQR=13, T0 HIT-6 Me=66 IQR=7, T1 HIT-6 Me=63 IQR=13, T0 ASC-12 Me=8 IQR=5, T1 ASC-12 Me=6 IQR=7. Associated symptoms changed as follow: T0 FSS Me=48 IQR=19, T1 FSS Me=33 IQR=23, T0 GAD-7 Me=10 IQR=8, T1 GAD-7 Me=9 IQR=7, T0 PHQ-9 Me=10 IQR=11, T1 PHQ-9 Me=7 IQR=7. After two injection cycles with OnabotulinumtoxinA, a statistically significant reduction was found in: a) frequency (p = 0.001; r = 0.51); b) acute medicines consumption/month (p = 0.001; r = 0.54); c) FSS score (p = 0.009; r = 0.41).

## Conclusions

OnabotulinumtoxinA resulted well tolerated and effective in reducing not only frequency and acute medication use, but also fatigue in our population of chronic refractory migraineurs.

Written informed consent to publication was obtained from the patient(s).

## References

[1] Schulman E (2013). Refractory migraine-a review. Headache.

[2] Aurora SK, Winner P (2011). OnabotulinumtoxinA for treatment of chronic migraine: pooled analyses of the 56-week PREEMPT clinical program. Headache.

